# Molecular Basis for a Lack of Correlation between Viral Fitness and Cell Killing Capacity

**DOI:** 10.1371/journal.ppat.0030053

**Published:** 2007-04-13

**Authors:** Mónica Herrera, Juan García-Arriaza, Nonia Pariente, Cristina Escarmís, Esteban Domingo

**Affiliations:** Centro de Biología Molecular “Severo Ochoa” (CSIC-UAM), Cantoblanco, Madrid, Spain; The Scripps Research Institute, United States of America

## Abstract

The relationship between parasite fitness and virulence has been the object of experimental and theoretical studies often with conflicting conclusions. Here, we provide direct experimental evidence that viral fitness and virulence, both measured in the same biological environment provided by host cells in culture, can be two unrelated traits. A biological clone of foot-and-mouth disease virus acquired high fitness and virulence (cell killing capacity) upon large population passages in cell culture. However, subsequent plaque-to-plaque transfers resulted in profound fitness loss, but only a minimal decrease of virulence. While fitness-decreasing mutations have been mapped throughout the genome, virulence determinants—studied here with mutant and chimeric viruses—were multigenic, but concentrated on some genomic regions. Therefore, we propose a model in which viral virulence is more robust to mutation than viral fitness. As a consequence, depending on the passage regime, viral fitness and virulence can follow different evolutionary trajectories. This lack of correlation is relevant to current models of attenuation and virulence in that virus de-adaptation need not entail a decrease of virulence.

## Author Summary

Virulence expresses the harm that parasites inflict upon their hosts. Many studies have addressed the basis of virulence and its effect on host and parasite survival. It has generally been accepted that one of the components of parasite virulence is fitness, or the capacity of the parasite to multiply in its host. Some models have equated virulence with fitness. In the present study, we use foot-and-mouth disease virus (FMDV) to document that virulence and fitness—measured in the same biological environment provided by cells in culture—can be unrelated traits. This has been achieved by multiplying the virus in a manner that mutations accumulated in its genome. Mutations decreased fitness dramatically, but not virulence. Chimeric and mutant viruses were constructed to show that virulence is influenced by only some of the FMDV genes, while fitness is influenced by the entire genome. For this reason, virulence is more robust (“resistant”) than fitness to the effects of deleterious mutations. The fact that virulence can be unrelated to fitness has implications for the design of anti-viral vaccines because it suggests that it may be possible to design high fitness, low virulence strains to stimulate the host immune response. Furthermore, in modelling studies it cannot be assumed that virulence is equal to fitness.

## Introduction

The relationship between fitness and virulence is an unsettled question, and sometimes fitness is considered a component of the virulence phenotype of parasites. RNA viruses are ideal systems to address this important question because of their high mutability and fecundity, which result in a potential for rapid evolution, and also because of the availability of quantitative fitness and virulence assays.

RNA viruses replicate as complex and dynamic mutant spectra, termed viral quasispecies. Key to quasispecies dynamics are mutation rates in the range of 10^−3^ to 10^−5^ substitutions per nucleotide copied, and competition among continuously arising variant genomes [1–4], which prompt rapid movements in sequence space, with corresponding changes of position in the fitness landscape [5]. Indeed, large population passages of RNA viruses in cell culture permit competitive optimization of mutant distributions that generally result in fitness gain [6,7], while repeated bottleneck events (experimentally realized as plaque-to-plaque transfers) lead to random accumulation of deleterious mutations (operation of Muller's ratchet [8]) and result in average fitness decreases [9–13]. Fitness recovery of low fitness foot-and-mouth disease virus (FMDV) clones occurs mainly with introduction of mutations along the genome, with very few true reversions.

An understanding of the consequences of fitness variation for viral virulence is a key question for viral pathogenesis and evolution. Here, we approach this issue with FMDV, an important viral pathogen in veterinary medicine [14], and one that fully participates of quasispecies dynamics. Our laboratory has characterized multiple FMDV variants that derive from one original biological clone, and that occupy widely different fitness levels when replicating in a defined environment in cell culture. We define *fitness* as the replication capacity of a mutant FMDV, relative to a reference FMDV, in direct growth-competition upon coinfection of baby hamster kidney 21 (BHK-21) cells [15–17]. Fitness of FMDV in BHK-21 cells is a multigenic trait [7].

In the present study, we define *virulence* of FMDV as the capacity of the virus to kill BHK-21 cells under a standard set of cell culture conditions [18]. Thus, the FMDV–BHK-21 system offered a means to investigate in a direct and comparative fashion the relationship between fitness and virulence of a virus, measured in the same biological environment provided by BHK-21 cells. We describe the behavior of an FMDV clone (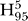 ), which has a history of repeated serial plaque-to-plaque transfers in BHK-21 cells [11], that attained a very low fitness value relative to its parental reference virus (C-S8c1), and yet, its virulence for BHK-21 cells was significantly higher than that of C-S8c1. A comparative study of the capacity to kill BHK-21 cells of chimeric FMDVs constructed with cDNA copies of the two parental FMDVs indicates that the enhanced virulence for BHK-21 cells of the low fitness clone is a polygenic trait, with participation of the regions encoding capsid proteins and non-structural proteins 2A, 2B, and 2C as virulence determinants. Three specific amino acid replacements in 2C have been identified as redundant virulence determinants of FMDV for BHK-21 cells. Thus, while large population passages of the virus resulted in a gain of both fitness and virulence, subsequent bottleneck passages resulted in a decrease of fitness but not of virulence.

The results suggest that fitness is very vulnerable to mutation in any genomic region. In contrast, because of the involvement of several (but not all) viral genes in virulence, and the redundant effect of three 2C substitutions, virulence is a more robust phenotypic trait than fitness, and less vulnerable to accumulation of mutations. Therefore, we provide direct evidence that viral fitness and capacity to kill cells can (in some cases) be unrelated traits. Furthermore, the relationship between fitness and virulence, of being either linked or unrelated traits, depends on the evolutionary history of the virus. This observation has implications for viral pathogenesis, and sheds light on models of virulence proposed on the basis of theoretical and experimental studies with cellular organisms.

## Results

### Inability of FMDV 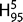 to Establish a Persistent Infection in BHK-21 Cells

**Figure 1:**
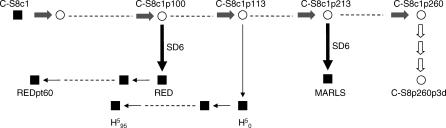
Schematic Representation of the Origin of the FMDVs Used in the Present Study C-S8c1 is the parental, reference biological clone of FMDV [19]. Biological clones are depicted as filled squares and uncloned populations as empty circles; p followed by a number indicates passage number. Large grey arrows indicate high MOI passages (1 to 5 PFU/cell); thin arrows describe the isolation of biological clones (virus from individual plaques) after dilution of virus and plating on BHK-21 cell monolayers; thick black arrows indicate the selection of mutant RED [20] and MARLS [21], which are resistant to neutralization by monoclonal antibody SD6; large empty arrows indicate low MOI passages carried out to derive the standard C-S8p260p3d genome from a bipartite, segmented form of the FMDV genome termed C-S8p260 [22]. The origin of viruses used in the present study is detailed in Materials and Methods.

Several biological clones and populations were obtained by passaging FMDV biological clone C-S8c1 [19–22] in BHK-21 cells, either as large population passages or plaque-to-plaque transfers (Figure 1). The biological clones and populations differed up to 236-fold in relative fitness (Table 1). The fitness differences found are expected from previous results on fitness gain upon large population passages of RNA viruses [6,7] and fitness decrease upon plaque-to-plaque (bottleneck) transfers [9–13]. The initial experiment was aimed at testing whether 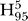 , because of its low fitness (0.11 times that of its parental C-S8c1 [12,23,24] [Table 1]), had an advantage in establishing a persistent non-cytopathic infection in BHK-21 cells as compared with its parental clone, C-S8c1 (Figure 1). A persistent FMDV infection is established by growing the cells that survive a standard cytolytic infection with FMDV [25]. Confluent monolayers of BHK-21 cells were infected either with C-S8c1 or with 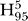 at a multiplicity of infection (MOI) of 0.02–0.1 plaque-forming units (PFU)/cell (2 × 10^6^ cells infected with 4 × 10^4^ −2 × 10^5^ PFU). Unexpectedly, at 24 h postinfection, the cells infected with 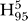 showed extensive cytopathology, and at 48 h postinfection, no surviving cells were observed. The frequency of surviving cells in parallel infections with C-S8c1 was 5 × 10^−3^–9 × 10^−3^, which is consistent with previous determinations [25]. No persistently infected BHK-21 cell cultures could be established with 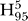 , despite several attempts. Thus, C-S8c1, which displays a 9-fold higher relative fitness than 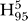 in BHK-21 cells, showed a capacity to kill BHK-21 cells that was at least 10^3^-fold lower than the killing capacity of 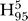 in the infectivity assay intended to establish a persistent FMDV infection.

**Table 1:**
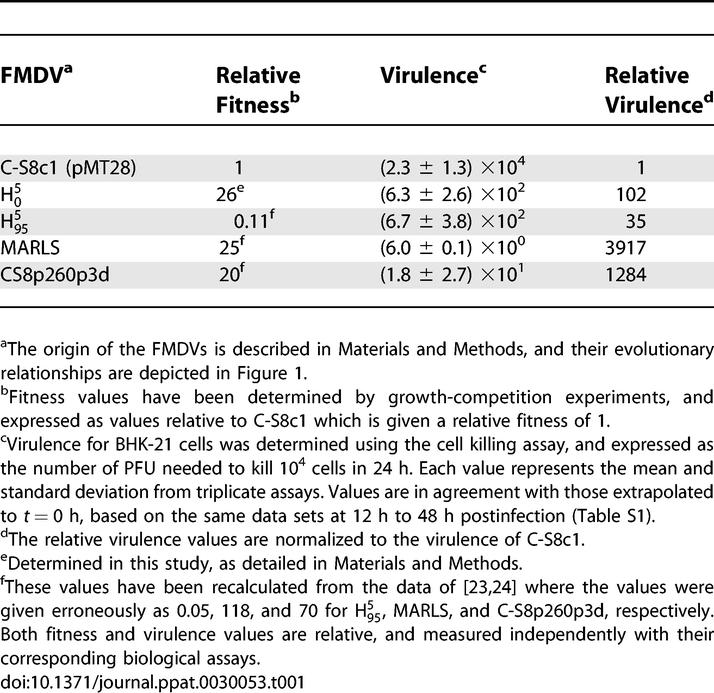
Values of Fitness and Virulence of FMDV Clones and Populations Analyzed in the Present Study

### FMDV Fitness and Capacity to Kill BHK-21 Cells May Not Be Positively Correlated

**Figure 2:**
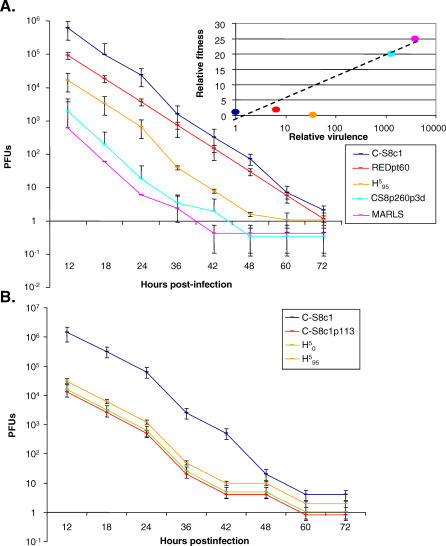
Killing of BHK-21 Cells by FMDVs (A) Time needed by C-S8c1, REDpt60, 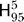 , C-S8p260p3d, and MARLS to kill 10^4^ BHK-21 cells as a function of the initial number of infectious units (PFU) added. The virulence assay is described in Materials and Methods. Each value represents the mean and standard deviation from triplicate assays. Inset: Relative fitness as a function of relative virulence values of FMDVs. The regression (discontinuous) line defined by C-S8c1, REDpt60, C-S8p260p3d, and MARLS is *y* = 3.026Ln(x) – 1.1028; *R* ^2^ = 0.9721. The regression line including 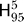 is *y* = 3.1458Ln(x) – 3.5111; *R* ^2^ = 0.8507 (not drawn). (B) Time needed by C-S8c1, C-S8c1p113, 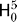 , and 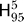 to kill 10^4^ BHK-21 cells as a function of the initial number of PFU. Each value represents the mean and standard deviation from triplicate assays. The viruses analyzed are described in Materials and Methods, and their evolutionary relationship is depicted in Figure 1. Virulence values are given in Tables 1, 2, and S1.

The capacity of 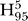 to kill BHK-21 cells despite its low fitness in BHK-21 cells led us to quantitatively examine the relationship between fitness of FMDV and its capacity to kill BHK-21 cells. To this aim, FMDV clones or populations were compared in a cell killing assay, consisting in determining the time required to kill 10^4^ BHK-21 cells as a function of the PFU added (described in Materials and Methods). The results (Figure 2A) indicate that over the time range of 12 h to 48 h postinfection, the number of PFUs needed to kill 10^4^ BHK-21 cells varied logarithmically as a function of time. Similar quantifications of relative virulence were obtained by measuring the PFU needed to kill 10^4^ cells in 24 h, and then by extrapolating the PFU values to 0 h postinfection (Tables 1 and S1). Virulence of 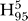 was 29 to 35 times higher than virulence of C-S8c1, despite the latter displaying a 9-fold higher fitness (Tables 1 and S1). The high virulence of 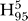 was not due to the plaque-to-plaque transfers, since a high virulence was also quantitated for its parental clone, 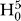 , and for population C-S8p113 (Figure 2B; Tables 1, 2, and S1). 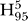 deviated from a line that correlated relative fitness of FMDV and the logarithm of cell killing capacity, as reflected in the decrease of the regression coefficient (*R* ^2^) (inset in Figure 2A). Probably, this deviation is due to the fact that 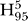 lost fitness due to plaque-to-plaque transfers, and the other viruses were not subjected to plaque-to-plaque transfers. On the other hand, virulence determinants were acquired during the large population passages done between C-S8c1 and C-S8c1p113. The 29- to 35-fold higher virulence of 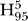 with respect to C-S8c1 (Tables 1 and S1), despite its low fitness, indicates that viral fitness and virulence can be two unrelated traits.

**Table 2:**
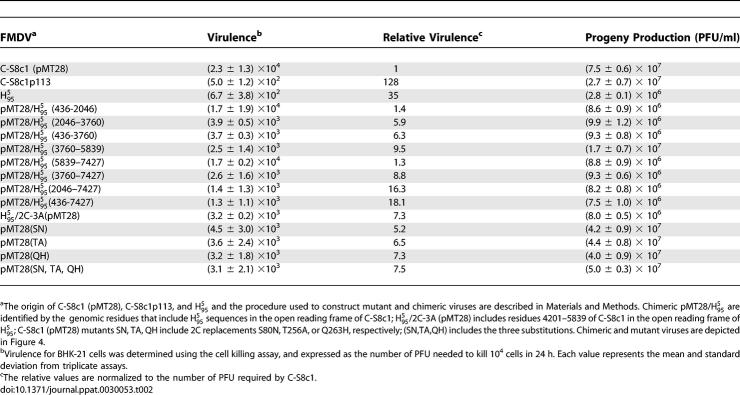
Virulence and Progeny Production by Chimeric C-S8c1 (pMT28)/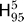 FMDVs and C-S8c1 (pMT28) with Amino Acid Substitutions in 2C

### Mapping Virulence Determinants in the 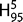 Genome

**Figure 3:**
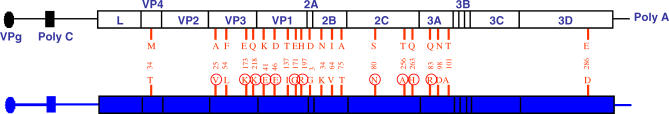
Amino Acid Substitutions Found in the FMDV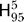 Genome as Compared to FMDV C-S8c1 The FMDV C-S8c1 genome (8,115 residues excluding the internal poly(C) and the 3′ poly(A)) composed of the 5′ and 3′ UTRs (lines) and coding regions (boxes), which include protease L, structural proteins (VP4, VP2, VP3, and VP1), and non-structural proteins (2A, 2B, 2C, 3A, 3B, 3C, and 3D). Genomic regions are based in [14] and references therein; VPg is the protein (3B) covalently linked to the 5′ end of the RNA, poly(C) is the internal polyribocytidylate tract, and poly(A) is the 3′ terminal polyadenylate tract. The FMDV C-S8c1- and 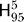 -coding regions are represented in white and blue, respectively. Amino acids in 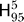 that differ from the corresponding ones in C-S8c1 are indicated in red. Replacements found also in the 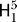 genome are encircled. Numbering of amino acids for each individual protein is as in [11].

**Figure 4:**
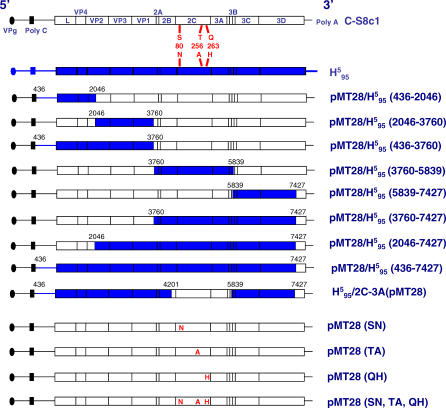
Scheme of the FMDV Genome, Chimeric Viruses, and 2C Mutants The C-S8c1 and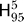 regulatory regions are represented as black and blue lines, respectively; the corresponding coding regions are represented as white and blue boxes, respectively. pMT28 refers to the plasmid that encodes infectious RNA transcripts with the C-S8c1 nucleotide sequence [22]. Chimeric viruses are colored blue and named with the first and the last nucleotide that correspond to the 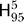 genomic region. Chimera 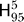 /2C-3A(pMT28) includes 2C, 3A, and parts of 2B and 3B of pMT28 in the background of residues 436 to 7427 of 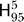 . Amino acid substitutions in protein 2C are indicated in red. The residue numbering of the FMDV genome is as in [11]. Procedures for the construction of chimeric viruses and 2C mutants are described in Materials and Methods.

**Figure 5:**
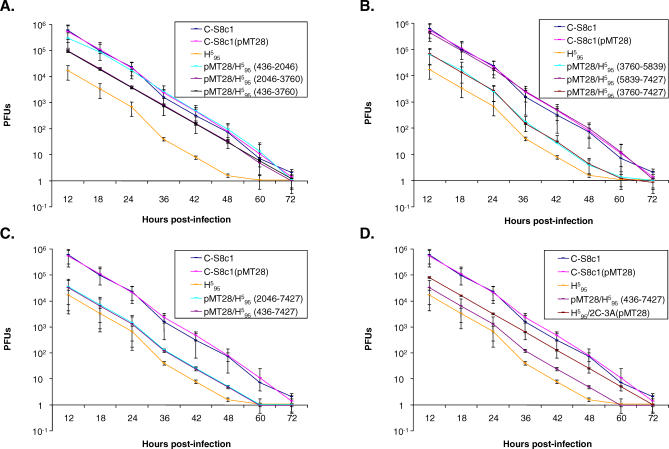
Killing of BHK-21 Cells by Recombinant FMDV Chimeras Time needed to kill 10^4^ BHK-21 cells as a function of the initial PFU of the following viruses: (A) C-S8c1, C-S8c1(pMT28),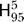 , pMT28/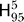 (436-2046), pMT28/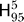 (2046–3760), and pMT28/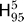 (436-3760). (B) C-S8c1(pMT28), 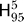 , pMT28/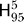 (3760–5839), pMT28/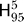 (5839–7427), and pMT28/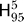 (3760–7427). (C) C-S8c1(pMT28), 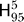 , pMT28/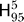 (2046–7427), and pMT28/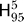 (436-7427). (D) C-S8c1(pMT28), 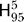 , pMT28/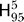 (436-7427), and 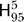 /2C-3A(pMT28). Each value represents the mean and standard deviation from triplicate assays. The viruses analyzed are described in Materials and Methods; C-S8c1(pMT28) is C-S8c1 expressed as an infectious transcript and rescued by cell transfection. Virulence values are given in Tables 2 and S1.

The comparison of the consensus nucleotide sequence of the 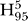 genome with that of C-S8c1 revealed a total of 47 mutations (Table S2), leading to 21 amino acid replacements affecting structural and non-structural proteins (Figure 3). To identify the genomic regions associated with the increased virulence of 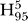 with respect to C-S8c1, we measured the BHK-21 cell killing capacity of nine chimeric viruses rescued from constructs obtained by introducing fragments of cDNA of the 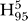 genome into plasmid pMT28, which encodes infectious C-S8c1 RNA [21] (Figure 4). The results (Figure 5; Tables 2 and S1) show that several genomic regions contribute to the virulence of 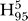 for BHK-21 cells, and that the major contributors map within genomic positions 2046 to 3760 (residues encoding part of VP2, VP3, and part of VP1, Figure 5A) and 3760 to 5839 (residues encoding 2A, 2B, 2C, and 3A, Figure 5B). The results exclude the internal ribosome entry site and the 3C- and 3D-coding regions as significant virulence determinants of 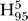 for BHK-21 cells (virulence of the relevant chimeric viruses ≤ 2.5, relative to C-S8c1; Tables 2 and S1). Infectious progeny production by each chimeric virus was intermediate between the production of the parental viruses pMT28 and 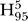 , with no significant differences that could be correlated with virulence (Table 2).

### Non-Structural Protein 2C Is a Determinant of the Virulence of FMDV for BHK-21 Cells

**Figure 6:**
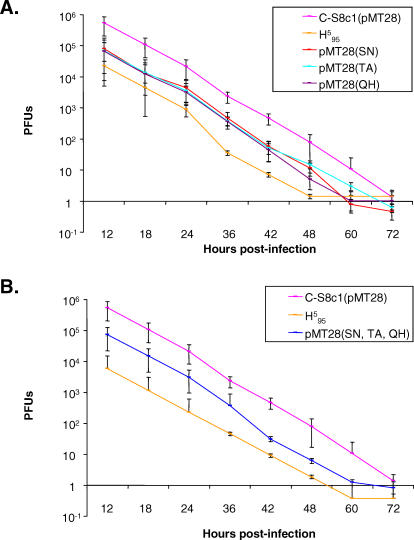
Killing of BHK-21 Cells by FMDV Mutants Time needed to kill 10^4^ BHK-21 cells as a function of the initial number of PFU of the following viruses: (A) C-S8c1(pMT28),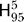 , pMT28 (SN), pMT28 (TA), and pMT28 (QH). (B) C-S8c1(pMT28), 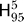
_,_ and pMT28 (SN, TA, QH). Each value represents the mean and standard deviation from triplicate assays. The viruses analyzed are described in Materials and Methods. Virulence values are given in Tables 2 and S1.

Amino acid substitutions in human rhinovirus protein 2C promoted cytopathology for mouse L cells [26]. Remarkably, 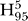 shares with other FMDV clones and populations, notably, MARLS and C-S8p260p3d (the two viruses showing the highest virulence for BHK-21 cells; Figure 2A; Tables 1 and S1), three amino acid substitutions in 2C: S80N, T256A, and Q263H. In addition, MARLS and CS8p260p3d include replacement M283V in 2C, relative to C-S8c1 [27,28]. To test whether any (or a combination) of the three shared amino acid substitutions in 2C contributed to the increased virulence of FMDV, each of the mutations was introduced individually into plasmid pMT28 by site-directed mutagenesis, as described in Materials and Methods. Transcripts of the three mutants, termed pMT28 (SN), pMT28 (TA), and pMT28 (QH) (Figure 4), were used to transfect BHK-21 cells, and the viruses obtained were tested with the BHK-21 cell killing assay. Viruses having any of the substitutions in 2C have a virulence intermediate between that of C-S8c1 and 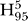 (Figure 6A). To test whether the combination of the three substitutions in 2C could produce an additional increase of virulence, the three mutations were introduced in pMT28 to rescue the triple mutant pMT28 (SN, TA, QH) (Figure 4). The results (Figure 6B) show that the virulence of the triple 2C mutant is similar to the virulence of the individual 2C mutants. The 2C mutations did not significantly affect the infectious progeny production (Table 2). A testable prediction of this result is that the introduction of the wild-type 2C-3A-coding region in the genetic background of 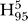 should produce a virus with lower virulence than 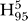 . Indeed, the results with such a chimeric virus (Figure 5D) indicate that the presence of the 2C- and 3A-coding region as the only genetic region of the pMT28 in the genetic background of 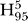 resulted in an FMDV with a 2.4- to 4.8-fold lower virulence than 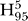 . We conclude that mutations in 2C contribute to virulence of FMDV for BHK-21 cells.

Thus, a virus that evolves towards low fitness levels due to the operation of Muller's ratchet may nevertheless maintain its capacity to kill the same cells in which it displays low fitness. In FMDV, the enhanced capacity to kill BHK-21 cells was multigenic, including participation of non-structural protein 2C with three amino acid substitutions acting in a redundant fashion. In conclusion, the results provide a molecular interpretation of why fitness and virulence of an animal virus can follow disparate evolutionary trajectories, culminating in two unrelated traits.

## Discussion

The capacity of a virus to kill cells is probably influenced by several steps in the virus life cycle, including receptor affinity (which may trigger signalling pathways and alter cell functions) and intracellular viral replication that may lead to metabolic alterations such as transcriptional or translational shut-off [29]. A parallel increase of virulence and fitness as a virus improves its adaptation to a host cell type is expected, since a key parameter that should contribute to the fitness level of a cytophatic virus is the accumulation of infectious particles and its release from cells, which are events often associated with cell killing. This expectation was fulfilled in our experiments (increase of both fitness and cell killing following large population passages of C-S8c1 [Figure 2]), and also in other virus–host systems. In a comparison of two genetically divergent isolates of the whispovirus white spot syndrome virus, whose virulence for the shrimp host Penaeus monodon was measured by in vivo cumulative mortality rates, virulence correlated with competitive fitness in vivo [30]. The onset of type 1 diabetes by coxsackievirus B strains was linked to the viral replication rate and to the infectious dose [31]. In engineered alphavirus replicons, a direct correlation between the level of RNA replication and cytopathogenicity was observed [32]. At an epidemiological level, a greater replicative fitness of historical versus current human immunodeficiency virus type 1 (HIV-1) isolates was taken as evidence of HIV-1 attenuation over time, assuming a direct connection between fitness and virulence [33]. In vivo, viral fitness may vary among specific organs, and virulence may be affected only when fitness for some specific target tissues is affected [34].

Replicative fitness is, however, but one of several factors which influence the progression of a viral infection in vivo. In a comparative analysis, R5-tropic and X4-tropic clones of HIV-1 showed similar replication capacity in mitogen-activated T cells. However, X4 clones were transferred more efficiently than R5 clones from dendritic cells to CD4(+) T cells, a fact that can contribute to the competitive advantage of X4 viruses in AIDS patients [35]. Simian immunodeficiency virus SIVmac239 infects both the sooty mangabey and the rhesus macaque, reaching high viral loads in both hosts, yet it is only virulent for the rhesus macaque [36]. Deviations of a positive correlation between viral fitness and virulence were observed also in the plant viruses cucumber mosaic virus [37] and barley stripe mosaic virus [38]. A study of the effect of lysis timing on bacteriophage fitness revealed that a strain with an intermediate lysis time had the highest fitness [39]. The time of transmission may also affect virulence. Nuclear polyhedrosis virus transmitted early to its host, the moth *Lymantria dispar,* was more virulent than virus transmitted late, although the latter was more productive because the virus could use more host tissue for replication [40]. In a study of the susceptibility of North American and non–North American breeds of *Lymantria* to several isolates of the fungus *Entomaphaga maimaiga,* mortality was scored in all cases. However, virulence of the fungus, quantitated by the time of death of *Lymantria,* was, in some cases, inversely proportional to fitness, quantitated by fungal reproduction in the moth [41]. In all these cases, the molecular basis of the lack of positive correlation between fitness and virulence is not understood.

### Model for a Lack of Correlation between Viral Fitness and Virulence

The results with FMDV clones H^5^ have documented that both fitness-enhancing and virulence-enhancing mutations can be incorporated in the viral genome in such a fashion that subsequent fitness-decreasing mutations associated with bottleneck (plaque-to-plaque) transfers produce only minimal effects on virulence (Figure 2). The dissection of accompanying molecular events, achieved through quantification of virulence of recombinant and mutant genomes (Tables 2 and S1), provides an interpretation of the lack of positive correlation between virulence and fitness. Multiple fitness-decreasing mutations occur in the course of plaque-to-plaque transfers, distributed throughout the FMDV genome [11]. In contrast, determinants of virulence for BHK-21 cells are multigenic, but concentrated mainly in some FMDV genomic regions. Similar multigenic but discrete virulence determinants have been described also in other virus–host systems [42,43]. To decrease virulence, mutations occurring randomly in the course of plaque-to-plaque transfers should affect specific genomic sites, and this will occur with a lower probability than fitness-decreasing mutations, which can hit any of the multifunctional picornaviral proteins and regulatory regions [11]. This model is reinforced by the observation that three amino acid substitutions in 2C (S80N, T256A, and Q263H) had a similar effect in enhancing FMDV virulence, and the three mutations in the same genome had an effect comparable to each mutation individually (Figure 6; Tables 2 and S1). It is not clear what the basis of the contribution of 2C to virulence for BHK-21 could be. 2C is involved in RNA synthesis and contains a nucleotide-binding domain, although none of the substitutions found in 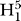 and 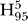 lie within such a domain. An unlikely triple reversion would be required to eliminate the virulence-enhancing effect of the three mutations in 2C. We propose that a higher robustness of the FMDV genome with regard to virulence for BHK-21 cells, rather than to replicative fitness in the same cells, underlies the different trajectories followed by fitness and virulence upon subjecting the virus to repeated bottleneck transfers. Obviously, we cannot exclude that parameters of the virus life cycle, other than fitness as measured in our experiments, could correlate with virulence for BHK-21.

The comparative analysis of FMDV clones and populations shows that shifts in virulence can occur even through the evolution of a single viral clone (C-S8c1), with its restricted genetic diversity prompted by different replication regimes in the same host cells, which also have a clonal origin (see Materials and Methods). We conjecture that the demonstration that fitness and virulence can follow different evolutionary courses has been possible thanks to the consequences of the extreme passage regimes to which the viral populations were subjected: competitive evolution of an ample mutant spectra during repeated large population passages, and accumulation of deleterious (with regard to fitness, but not with regard to virulence) mutations upon plaque-to-plaque transfers (predominance of genetic drift and operation of Muller's ratchet) [12,15].

### Implications for Models of Virulence

It must be emphasized that fitness and virulence are relative values that pertain to a defined physical and biological environment. Virulence determinants of FMDV, identified here for BHK-21 cells, need not apply to virulence for the natural animal hosts of FMDV [44]. However, the observation of a lack of correlation between fitness and virulence in a FMDV clone is relevant to current models of attenuation and virulence, since it shows that more virulent forms of a virus need not have a reproductive advantage, and that viral virulence is not necessarily a byproduct of viral fitness. Even if virulence is regarded as an unavoidable consequence of parasite adaptation [45], virus de-adaptation (fitness loss) need not entail a decrease of virulence.

Most current definitions of virulence include both the ability of the pathogen to multiply and to cause harm to its host; some authors, however, assume a direct relationship between fitness and capacity to produce disease [46–48]. In relating the results with FMDV to general models of virulence in host–parasite systems, it must be considered that in the FMDV system, evolution of the host BHK-21 cells could not influence FMDV evolution, because clonal cell populations with a controlled passage history were supplied in constant numbers at each infection event (see Materials and Methods). Therefore, changes in host density, or mobility, as well as pathogen survival in the external environment, all of which are relevant parameters in virulence models [48,49], cannot play a role in our system. A consistent finding in serial passage experiments is that virulence of a parasite increases with passage number in a new host [50]. The results with FMDV infecting BHK-21 cells cytolytically imply that the increase of virulence can be conditioned to the history of passage regimes undergone by a virus.

The invariance of BHK-21 cells in the course of serial cytolytic passages of FMDV is in contrast with the parallel system consisting of BHK-21 cells persistently infected with FMDV C-S8c1 [25], in which the cells are passaged and coevolve with the resident virus [51]. Host–virus coevolution has generally favored a decrease of viral virulence in the field, a classical example being myxoma virus and myxomatosis in rabbits [52].

Our comparison of FMDV clones did not provide evidence of clones with high fitness and low virulence, which, with regard to natural hosts, is an aim of biomedicine to obtain vaccine strains. Yet, the existence of specific mutations that differentially affect fitness and virulence opens the way to engineer candidate vaccine strains unable to kill the host, while maintaining replicative competence. Virulence is, however, a feature of the host–parasite relationship [46], and the mutations needed to impair virulence are expected to be host-dependent [53,54].

## Materials and Methods

### Cells, viruses, and infections.

The BHK-21 cells used in the present study were cloned by end-point dilution, followed by preparation of a cell stock from a single cell; they were passaged a maximum of 30 times before being used for FMDV infection [25,51]. Procedures for cell growth, infection of BHK-21 cell monolayers with FMDV in liquid medium, and plaque assays in semi-solid agar medium were carried out as previously described [11,19,25,27]. Mock-infected cells were handled in parallel in all infectivity and plaque assays to monitor absence of viral contamination. The FMDVs used in the present study (Figure 1) are (i) the reference clone C-S8c1, which has been assigned a relative fitness of 1.0 [11]. (ii) MARLS, a monoclonal antibody escape mutant isolated from population C-S8c1p213 [55]; MARLS has a fitness of 25 relative to C-S8c1 [24]. (iii) C-S8p260p3d, a standard FMDV virus rescued by low MOI passage of C-S8p260. The latter is a virus that evolved by passage of C-S8c1 at a high MOI, which resulted in dominance of two defective FMDV genomes (both including internal deletions) that were infectious by complementation, in the absence of standard virus [22,24,28]; C-S8p260p3d has a relative fitness of 20 [24]. (iv) REDpt60, obtained after 60 successive plaque-to-plaque transfers of RED (a monoclonal antibody escape mutant isolated from population C-S8c1p100) [20]; REDpt60 has a fitness of 1.9 relative to C-S8c1. (v) C-S8c1p113, a viral population obtained after 113 serial cytolytic passages of C-S8c1 at a high MOI in BHK-21 cells (2 × 10^6^ BHK-21 cells infected with the virus contained in 200 μl of the supernatant from the previous infection). (vi) Clone 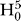 , a biological clone isolated from population C-S8c1p113 [11]; its relative fitness is 26 (unpublished data). (vii) Clone 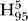 , obtained after 95 successive plaque-to-plaque transfers of 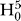 [11]; its relative fitness is 0.11 [23].

### Cell killing assay.

The capacity of FMDV to kill BHK-21 cells was measured as previously described [18,22]. The assay consists in determining the minimum number of PFU required to kill 10^4^ BHK-21 cells after variable times of infection. The assay was performed in M96 multiwell plates with monolayers of 10^4^ BHK-21 cells per well infected with serial dilutions of virus. At different times postinfection, cells were fixed with 2% formaldehyde and stained with 2% crystal violet in 2% formaldehyde. Results are expressed as the logarithm of the number of PFUs needed for complete cell killing (as judged by cell staining with crystal violet, with series of control wells with known numbers of cells) as a function of time postinfection [18,22].

### Determination of relative fitness of 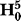 .

The relative fitness of FMDV 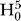 was determined by growth competition in BHK-21 cells as previously described [7,10,11,24,56]. FMDV 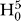 was mixed with appropriate proportions of 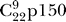 , which was used as reference virus. This virus has a fitness 8.5-fold higher than that of the reference clone of C-S8c1 in BHK-21 cells [7,10,11,24,56]. Four serial infections were carried out at MOI of 0.1 PFU/cell. The proportion of the two competing genomes at different passages was determined by real-time reverse transcription (RT)–PCR, employing primers 5531wtnew and 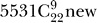 , which are able to discriminate FMDV 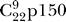 RNA from 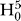 RNA. The nucleotide sequences of the primers will be provided upon request. The fitness vector obtained for 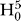 corresponded to the equation *y* = 0,0206e^1,1074x^; *R* ^2^ = 0.9507. The antilogarithm (base e) of the vector slope is the fitness of the assayed virus relative to the reference virus [56].

### cDNA synthesis, molecular cloning, and nucleotide sequencing.

Viral RNA was extracted by treatment with Trizol as previously described [57]. Reverse transcription of FMDV RNA was carried out with avian myeloblastosis virus reverse transcriptase (Promega, http://www.promega.com) or Transcriptor reverse transcriptase (Roche, http://www.roche.com), and PCR amplification was performed by using either Ampli-Taq polymerase (PerkinElmer, http://las.perkinelmer.com) or an Expand High Fidelity polymerase system (Roche), as instructed by the manufacturers. The FMDV genome-specific oligonucleotide primers used have been previously described [22,58]. In all RT-PCR amplifications, negative amplification controls, including all reaction components except template RNA, were run in parallel to monitor absence of contamination.

Chimeric viruses containing selected regions of 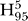 in the genetic background of C-S8c1 (Figure 4) were obtained by replacing the corresponding DNA fragment of pMT28 by a cDNA copy of 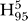 RNA, using specific restriction sites. To obtain pMT28/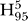 (436-2046), a chimera that included nucleotides 436 to 2046 of 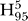 (the residue numbering of the FMDV genome is as in [11]), 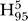 RNA was amplified by RT-PCR using primers NR2 and JH2, and the cDNA was digested with Hpa I (position 436) and Xba I (2046), and ligated into pMT28 DNA digested with the same enzymes. To obtain pMT28/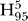 (2046–3760), 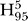 RNA was amplified by RT-PCR using primers 2R1New and pU, and then the cDNA was digested with Xba I (2046) and Avr II (3760). To obtain pMT28/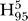 (3760–5839), 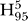 RNA was amplified by RT-PCR using primers 3R2New and 3CD1, and then the cDNA was digested with Avr II (3760) and Rsr II (5839). To obtain pMT28/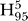 (5839–7427), 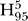 RNA was amplified by RT-PCR using primers 5531 wt new and C-Not-Pol, and then the cDNA was digested with Rsr II (5839) and Bam HI (7427). To obtain pMT28/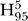 (436-3760), 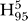 RNA was amplified by RT-PCR using primers NR2 and JH2, and then the cDNA was digested with Hpa I (position 436) and Xba I (2046), and ligated into pMT28/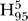 (2046–3760) DNA digested with the same enzymes. To obtain pMT28/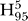 (3760–7427), 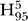 RNA was amplified by RT-PCR using primers 3R2New and 3CD1, and then the cDNA was digested with Avr II (3760) and Rsr II (5839), and ligated into pMT28/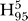 (5839–7427) DNA digested with the same enzymes. To obtain pMT28/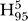 (2046–7427), 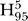 RNA was amplified by RT-PCR using primers 2R1New and pU, and then the cDNA was digested with Xba I (2046) and Avr II (3760), and ligated into pMT28/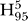 (3760–7427) DNA digested with the same enzymes. To obtain pMT28/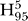 (436-7427), 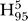 RNA was amplified by RT-PCR using primers NR2 and JH2; the cDNA was digested with Hpa I (position 436) and Xba I (2046), and ligated into pMT28/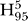 (2046–7427) DNA digested with the same enzymes. To obtain 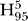 /2C-3A(pMT28), pMT28 was digested with Bgl II (4201) and Rsr II (5839), and a DNA fragment including wild-type 2C-3A-coding region was inserted into pMT28/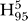 (436-7427) DNA digested with the same enzymes. DNA ligation, transformation of Escherichia coli DH5α, isolation of DNA from bacterial colonies, and characterization of DNA by restriction enzyme digestion were performed by standard procedures [59]. The primers used for molecular cloning and site-directed mutagenesis are described in Table S3.

To obtain FMDV C-S8c1 containing the mutations found in gene 2C of 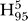 , plasmid pMT28 was subjected to site-directed mutagenesis using an oligonucleotide including the required nucleotide replacement, and 3R2New or 3CD1 as external oligonucleotide primer (Table S3; Figure 4). A DNA fragment termed A was obtained by subjecting plasmid pMT28 to site-directed mutagenesis using primers (reverse) mutSNu, mutTAu, and mutQHu (to introduce mutations S80N, T256A, and Q263H, respectively) and an external oligonucleotide primer (3R2New, forward). A DNA fragment termed B was obtained amplifying pMT28 with primers (forward) mutSNd, mutTAd, and mutQHd (to introduce mutations S80N, T256A, and Q263H, respectively) and an external oligonucleotide primer (3CD1, reverse). DNA fragments A and B, including the desired mutations, were recombined by shuffling PCR using equimolar amounts of DNA fragments and two external primers (3R2New and 3CD1). The DNA with the desired mutation(s) in the 2C gene was digested with Avr II (genomic position 3760) and Rsr II (position 5839), and cloned into pMT28 to generate pMT28 (SN), pMT28 (TA), and pMT28 (QH). To obtain pMT28 (SN, TA, QH), plasmid pMT28 (SN) was subjected to site-directed mutagenesis to introduce mutation T256A in a similar way as described above, and then, plasmid pMT28 (SN, TA) was subjected to site-directed mutagenesis to introduce mutation Q263H. All chimeric viruses and mutants were analyzed by nucleotide sequencing using Big Dye Terminator Cycle Sequencing kit (Abi Prism; PerkinElmer) and sequencer ABI373 as previously described [58]. Sequences were analyzed using DNASTAR 4.0 (http://www.dnastar.com), GeneDoc, and GCC (University of Wisconsin). Each sequence was determined at least twice, with products obtained using different oligonucleotide primers.

DNA from pMT28 or its recombinant and mutant derivatives was linearized with Nde I and transcribed with SP6 RNA polymerase as previously described [22,27]. Transcript RNA integrity and concentration were estimated by agarose gel electrophoresis, in parallel runs with known amounts of standard C-S8c1 RNA. BHK-21 cell monolayers (70% confluent, about 1 × 10^6^ cells) were transfected with RNA transcripts (1 μg RNA) using lipofectin as previously described [59]. Virus was collected from the culture supernatant at 72 h post-transfection. The virus obtained by transfection was passaged twice before using it in biological studies. RNA was extracted and sequenced to ascertain that the virus maintained the genomic structure and mutations of the initial transcript.

Consensus genomic nucleotide sequences of FMDV clones were obtained by RT-PCR amplification of virion RNA using specific primers [7,22,28].

## Supporting Information

Table S1 Calculation of Virulence of FMDV Variants by Extrapolation to 0 h Postinfection ^a^The origin of the FMDVs is described in Materials and Methods.
^b^Estimated relative virulence as the value extrapolated from the regression at 0 h postinfection.
^c^Regression lines with the data of 12 h to 48 h postinfection were obtained using the program Excel from the Microsoft Office package.
(46 KB DOC)

Table S2Mutations Found in the FMDV 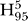 Genome as Compared to FMDV C-S8c1

^a^The first letter corresponds to the nucleotide found in the parental FMDV C-S8c1, and the number gives the nucleotide position in the C-S8c1 genome [11].
^b^The first amino acid is the one found in C-S8c1; amino acid residues are numbered independently for each protein. -, synonymous mutation; *, amino acid change in invariant positions of the FMDV genome according to [60].
^c^H clone in which the mutation appears for the first time [11,58].(88 KB DOC)

Table S3 Oligonucleotide Primers Used in Molecular Cloning and Site-Directed Mutagenesis ^a^The residue numbering of the FMDV genome is as in [11].
^b^Nucleotide replacements required for mutagenesis are underlined.
(44 KB DOC)

### Accession Numbers

The GenBank accession numbers for the C-S8c1, 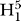 , 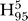 , CS8p260p3d, and MARLS genomic sequences are AJ133357, AM409190, AM409325, DQ409185, and AF274010, respectively. Nucleotide and amino acid sequences for picornaviruses can be found at http://www.iah.bbsrc.ac.uk/virus/picornaviridae/SequenceDatabase/3Ddatabase/3D.HTM.

## Abbreviations

BHK-21: baby hamster kidney 21
FMDV: foot-and-mouth disease virus
HIV-1: human immunodeficiency virus type 1
MOI: multiplicity of infection
PFU: plaque-forming units
RT-PCR: reverse transcription PCR
