# Supplementary material for: Molecular Basis for a Lack of Correlation between Viral Fitness and Cell Killing Capacity
Source: PLoS Pathog. 2007 Apr 13;3(4):e53. doi: 10.1371/journal.ppat.0030053 (PMC1851977; doi:10.1371/journal.ppat.0030053)
Supplement: Supplementary file 1 [file ppat.0030053.st001.doc]

**Table S1**

| **FMDV ^a^** | **Relative virulence ^b^**  **t = 0** | **Regression lines ^c^** | **R^2^** |
| --- | --- | --- | --- |
|  |  |  |  |
| C-S8c1 (pMT28) | 1 | y = 1E+07e^-0.2458x^ | 0.9985 |
| C-S8c1p113 | 55 | y = 178670e^-0.2402x^ | 0.9775 |
| MARLS | 4348 | y = 2278.5e^-0.1942x^ | 0.9183 |
| CS8p260p3d | 909 | y = 11483e^-0.2191x^ | 0.9505 |
| H^5^_95_ | 29 | y = 340218e^-0.2548x^ | 0.9995 |
| H^5^_0_ | 45 | y = 217682e^-0.2395x^ | 0.9776 |
| pMT28/H^5^_95_ (436-2046) | 2 | y = 4E+06e^-0.2165x^ | 0.9928 |
| pMT28/H^5^_95_ (2046-3760) | 10 | y = 983329e^-0.2108x^ | 0.9878 |
| pMT28/H^5^_95_ (436-3760) | 11 | y = 928447e^-0.2108x^ | 0.9878 |
| pMT28/H^5^_95_ (3760-5839) | 5 | y = 2E+06e^-0.2649x^ | 0.9966 |
| pMT28/H^5^_95_ (5839-7427) | 2 | y = 5E+06e^-0.2232x^ | 0.9933 |
| pMT28/H^5^_95_ (3760-7427) | 5 | y = 2E+06e^-0.2623x^ | 0.9985 |
| pMT28/H^5^_95_ (2046-7427) | 18 | y = 565229e^-0.239x^ | 0.9975 |
| pMT28/H^5^_95_ (436-7427) | 20 | y = 500391e^-0.2381x^ | 0.9973 |
| H^5^_95_/2C-3A(pMT28) | 12 | y = 798314e^-0.2108x^ | 0.9878 |
| pMT28(SN) | 10 | y = 1E+06e^-0.2358x^ | 0.9894 |
| pMT28(TA) | 10 | y = 1E+06e^-0.2303x^ | 0.9953 |
| pMT28(QH) | 10 | y = 1E+06e^-0.2494x^ | 0.9858 |
| pMT28(SN, TA, QH) | 5 | y = 2E+06e^-0.2536x^ | 0.9886 |
