# Supplementary material for: Molecular Basis for a Lack of Correlation between Viral Fitness and Cell Killing Capacity
Source: PLoS Pathog. 2007 Apr 13;3(4):e53. doi: 10.1371/journal.ppat.0030053 (PMC1851977; doi:10.1371/journal.ppat.0030053)
Supplement: Supplementary file 2 [file ppat.0030053.st002.doc]

**Table S2**

| **Genomic region** | **Mutation ^a^** | **Amino acid substitution ^b^** | **H clone ^c^** |
| --- | --- | --- | --- |
|  |  |  |  |
| S fragment | C-32-A | - | H^5^_80_ |
|  | C-64-U | - | H^5^_1_ |
|  | C-247-U | - | H^5^_1_ |
| Pseudoknot | G-476-A | - | H^5^_1_ |
|  | A-492-G | - | H^5^_80_ |
|  | G-504-U | - | H^5^_80_ |
|  | C-509-U | - | H^5^_80_ |
| IRES | A-809-G | - | H^5^_95_ |
|  | U-856-C | - | H^5^_1_ |
| VP4 | U-1742-C | M 34 T * | H^5^_80_ |
|  | A-1851-G | - | H^5^_30_ |
| VP3 | C-2624-U | A 25 V | H^5^_1_ |
|  | U-2710-C | F 54 L | H^5^_80_ |
|  | G-3067-A | E 173 K | H^5^_1_ |
|  | C-3120-U | - | H^5^_80_ |
|  | C-3202-A | Q 218 K | H^5^_1_ |
| VP1 | A-3328-G | K 41 E | H^5^_1_ |
|  | G-3342-A | - | H^5^_30_ |
|  | U-3345-A | D 46 E | H^5^_1_ |
|  | C-3617-U | T 137 I | H^5^_95_ |
|  | U-3660-C | - | H^5^_30_ |
|  | A-3719-G | E 171 G | H^5^_1_ |
|  | A-3797-G | H 197 R | H^5^_1_ |
| 2A | A-3842-G | D 3 G | H^5^_30_ |
|  | U-3864-C | - | H^5^_80_ |
| 2B | C-3891-U | - | H^5^_80_ |
|  | A-3933-G | - | H^5^_80_ |
|  | C-3984-A | N 34 K | H^5^_80_ |
|  | A-4072-G | I 64 V * | H^5^_80_ |
|  | G-4105-A | A 75 T * | H^5^_80_ |
| 2C | G-4583-A | S 80 N | H^5^_1_ |
|  | U-4812-C | - | H^5^_95_ |
|  | U-5077-C | - | H^5^_80_ |
|  | A-5110-G | T 256 A | H^5^_1_ |
|  | G-5133-C | Q 263 H | H^5^_1_ |
| 3A | U-5454-C | - | H^5^_1_ |
|  | A-5546-G | Q 83 R | H^5^_1_ |
|  | A-5590-G | N 98 D | H^5^_30_ |
|  | A-5599-G | T 101 A | H^5^_80_ |
| 3C | U-6306-C | - | H^5^_1_ |
| 3D | U-6702-C | - | H^5^_95_ |
|  | U-6724-C | - | H^5^_80_ |
|  | A-6753-G | - | H^5^_80_ |
|  | U-6789-C | - | H^5^_80_ |
|  | G-7467-U | E 286 D | H^5^_80_ |
|  | G-7554-U | - | H^5^_1_ |
| 3’ UTR | G-8042-C | - | H^5^_30_ |
