# Supplementary material for: Molecular Basis for a Lack of Correlation between Viral Fitness and Cell Killing Capacity
Source: PLoS Pathog. 2007 Apr 13;3(4):e53. doi: 10.1371/journal.ppat.0030053 (PMC1851977; doi:10.1371/journal.ppat.0030053)
Supplement: Supplementary file 3 [file ppat.0030053.st003.doc]

**Table S3**

| **Genomic**  **region** | **Primer** | **Sequence (5’to 3’)** | **Orientation** | **Position^a^** |
| --- | --- | --- | --- | --- |
|  |  |  |  |  |
| Pseudoknot | NR2 | TAAGTTTTACCGTCTGTCCCG | sense | 368 |
| VP2 | 2R1New | ctggtctagagagacgcgcgttcatc | sense | 2042 |
|  | JH2 | GGGTTGATGAACTGGTGGGG | antisense | 2345 |
| VP3 | 3R2New | ctttgagctccggctacctgtg | sense | 3171 |
| 2A-2B | pU | GAAGGGCCCAGGGTTGGACT | antisense | 3888 |
| 2C | mutSNu | ttgaagaacgggaacgtccatattgc^b^ | sense | 4576 |
|  | mutSNd | cgttcccgttcttcaaacacacttgg^b^ | antisense | 4591 |
|  | mutTAu | gaagacacccacgccaatccagtggc^b^ | sense | 4098 |
|  | mutTAd | actggattggcgtgggtgtcttcaagtgc^b^ | antisense | 5120 |
|  | mutQHu | ggcaatgtttcactacgactgtgccc^b^ | sense | 5121 |
|  | mutQHd | ggcacagtcgtagtgaaacattgcc^b^ | antisense | 5145 |
|  | 5331wtnew | GTCATCATGATCCGCGA | sense | 5515 |
| 3C | 3CD1 | catgaccatcttttgcaggtcag | antisense | 6003 |
| 3D | C-Not-Pol | CCAattgtgatgtttggcggccgctgcgtcgccgcacacggcgttc | antisense | 8043 |
